# Functional fine-tuning between bacterial DNA recombination initiation and quality control systems

**DOI:** 10.1371/journal.pone.0192483

**Published:** 2018-02-22

**Authors:** Veronika Ferencziová, Gábor M. Harami, Julianna B. Németh, Tibor Vellai, Mihály Kovács

**Affiliations:** 1 Department of Biochemistry, ELTE-MTA “Momentum” Motor Enzymology Research Group, Eötvös Loránd University, Pázmány P. s. 1/c, Budapest, Hungary; 2 Department of Genetics, Eötvös Loránd University, Pázmány P. s. 1/c, Budapest, Hungary; Niels Bohr Institute, DENMARK

## Abstract

Homologous recombination (HR) is crucial for the error-free repair of DNA double-strand breaks (DSBs) and the restart of stalled replication. However, imprecise HR can lead to genome instability, highlighting the importance of HR quality control. After DSB formation, HR proceeds via DNA end resection and recombinase loading, whereas helicase-catalyzed disruption of a subset of subsequently formed DNA invasions is thought to be essential for maintaining HR accuracy *via* inhibiting illegitimate (non-allelic) recombination. Here we show that *in vitro* characterized mechanistic aberrations of *E*. *coli* RecBCD (resection and recombinase loading) RecQ (multifunctional DNA-restructuring helicase) mutant enzyme variants, on one hand, cumulatively deteriorate cell survival under certain conditions of genomic stress. On the other hand, we find that RecBCD and RecQ defects functionally compensate each other in terms of HR accuracy. The abnormally long resection and unproductive recombinase loading activities of a mutant RecBCD complex (harboring the D1080A substitution in RecB) cause enhanced illegitimate recombination. However, this compromised HR-accuracy phenotype is suppressed in double mutant strains harboring mutant RecQ variants with abnormally enhanced helicase and inefficient invasion disruptase activities. These results frame an *in vivo* context for the interplay of biochemical activities leading to illegitimate recombination, and underscore its long-range genome instability effects manifest in higher eukaryotes.

## Introduction

Homologous recombination (HR) is a biological process that occurs in all kingdoms of life, and provides the most efficient means for the error-free repair of DNA double-strand breaks (DSBs) and the restart of stalled DNA replication [1,2]. HR is mediated by the resection of DSB ends, producing 3’ single-stranded (ss) DNA overhangs that are substrates for the subsequent formation of recombinase nucleoprotein (RNP) filaments. RNP filaments catalyze invasion into, and homology search within, an undamaged homologous DNA template, leading to the formation of joint DNA molecules (JMs). Subsequent DNA synthesis and various pathways of JM processing ultimately lead to the restoration of the information content of the damaged DNA molecule by using information from the undamaged template [2,3].

In *E*. *coli*, RecBCD is the main enzyme complex catalyzing DSB end resection and coupled RecA recombinase loading, resulting in efficient production of RNP filaments on 3’ ssDNA overhangs [3]. Amino acid substitutions in the nuclease/RecA loading domain of the RecB subunit (e.g., D1080A, denoted as *recB1080*) lead to enhanced helicase (dsDNA unwinding) activity of the RecBCD complex, associated with compromised nuclease and RecA loading activities[4–6]. As a result, the mutant RecBCD complex produces abnormally long 5’ and 3’ overhangs that can only be partially filled with RecA [4]. Thus, the *recB1080* genotype is associated with a specific, well-characterized alteration in the initiation (resection/RNP formation) mechanism of bacterial HR [4, 6].

RecQ helicase, a member of the alternative RecF recombination pathway, has been implicated to play multiple important roles assisting DNA replication and HR-based repair [7]. In conjunction with RecJ exonuclease, RecQ can catalyze DNA end resection during the repair of single-strand gaps (SSGs) in DNA. Moreover, the ‘invasion disruptase’ function of RecQ is thought to be important for the quality control (i.e., accuracy) of HR, as RecQ can unwind short base-paired DNA intermediates formed between non-allelic DNA sequences that could lead to illegitimate recombination (IR) [7,8]. The dsDNA unwinding activity of RecQ also contributes to the restart of stalled replication and the completion of chromosomal replication [9–11]. The latter function of the eukaryotic homologs of RecQ is indicated by severe hereditary diseases associated with hyperrecombination, genome instability, accelerated aging and high cancer predisposition [12–14].

In our recent work we showed that RecQ exhibits characteristic complex biochemical activities besides the elementary dsDNA unwinding activity [15]. RecQ displays short-range repetitive shuttling (unwinding and rezipping) along DNA segments as well as directed disruption of DNA strand invasions, HR intermediates thought to be the key targets for HR quality control [15]. In addition, we identified engineered RecQ variants that perform enhanced dsDNA unwinding but specifically lack the complex shuttling and disruptase activities (see below). We showed that these properties together are detrimental for HR accuracy, and lead to IR frequencies elevated significantly over the wild-type (WT) level [15].

The above described specific, well-characterized alterations of RecBCD and RecQ activities provide an unprecedented opportunity to probe the poorly understood, but potentially crucially important, functional interplay between these enzymes in the repair of DNA lesions and in HR quality control. In the present study we quantitatively tested the phenotypic effects of these alterations, and their combination, on cell growth/survival under normal and genotoxic conditions, besides measuring HR accuracy in untreated cells. We find that the specific RecBCD and RecQ functional defects have cumulative effects on the survival of certain forms of genomic stress. Surprisingly, however, we show that the compromised HR accuracy resulting from aberrant RecBCD function is significantly ameliorated by specifically perturbing, but not by abolishing, RecQ activities. These results suggest that the activities of different HR enzymes have been adaptively co-optimized to their specific natural (WT) functional environment in order to serve efficient DNA lesion repair on one hand, and long-range genome stability (HR accuracy) on the other. The importance of a similar balance in higher eukaryotes is reflected in the association of similar functional aberrations with genome instability and cancer predisposition syndromes [16–18].

## Results

### Design and generation of *E*. *coli* strains

By using the RedET recombination system, we generated the following mutant *E*. *coli* strains (using the MG1655 strain as WT) expressing modified protein variants driven by their respective native promoters (**S1 Table**). *recB1080* harbors a RecB protein with amino acid (aa) substitution D1080A, which has been shown to cause enhanced helicase activity of the RecB^1080^CD complex, combined with impaired nuclease and RecA-loading activities [5]. Thus, RecB^1080^CD performs modified DSB processing that results in abnormally long 5’ and 3’ ssDNA overhangs that are only partially filled with RecA [6]. The recQ* strain harbors the Y555A substitution within RecQ’s Helicase-and-RNaseD-C-terminal (HRDC) domain, abolishing the ssDNA interaction of this domain [19]. In the *recQ-dH* and *recQ-dWH* strains the protein is C-terminally truncated after aa 523 and 413, respectively, thereby lacking the HRDC (*recQ-dH*) and winged-helix (WHD) plus HRDC domains (*recQ-dWH*) [15, 20].The RecQ* and RecQ-dH proteins exhibit enhanced DNA unwinding activity, but lack the shuttling activity characteristic of RecQ-WT, and RecQ-dH is further severely impaired in its directed invasion disruption activity [15, 20]. RecQ-dWH is a weak helicase that can be considered a loss-of-function variant for all DNA-restructuring activities [15]. The *ΔrecQ* strain is a null mutant expressing no RecQ helicase [15]. In addition, we generated double mutant strains for *recB1080* and each *recQ* gene variant to assess the interplay between RecBCD and RecQ activities.

In the experiments described below, we quantitatively determined how the above mutations affect cell growth and division under stress-free conditions, cell survival under different forms of genomic stress, and HR accuracy under stress-free conditions.

### The *recB1080* mutation, but not *recQ* defects, impairs cell division under stress-free conditions

**Fig 1:**
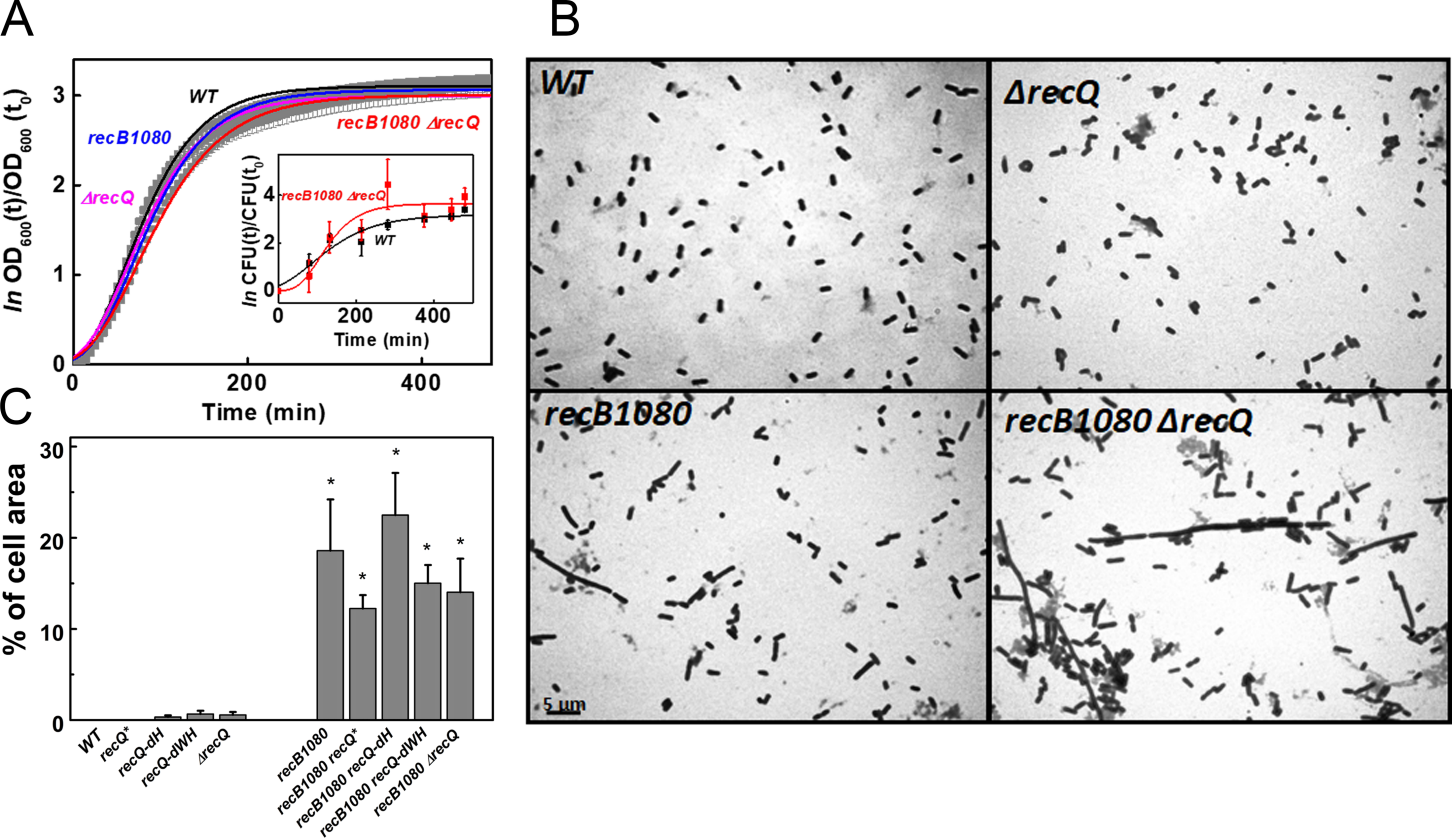
RecB and RecQ defects do not affect cell growth, whereas the *recB1080* mutation markedly impairs cell division under stress-free conditions. **(A)** Growth (*OD*_600_) curves of indicated *E*. *coli* strains, fitted by the modified Gompertz model (solid lines, **S1 Eq**) (21). Means ± SE for *n* = 5 biological replicates are shown. Error bars are within symbols where not visible. Inset: cell growth monitored via changes in CFU/ml over time (means ± SE for *n* = 3). Determined parameters and analysis results are shown in **S1 Fig** and **S2 Table**. **(B)** Example images of methylene blue stained cells (see also **A panel in S2 Fig**). **(C)** Fraction of the total cell area found in large elongated structures (with individual area > 4 μm^2^; see also **Panel D in S2 Fig**). Percentages for the number of large cells (> 4 μm^2^) and other determined parameters are listed in **S3 Table**. Means ± SE for *n* = 3 biological replicates are shown. Asterisks indicate significant difference from the WT value (ANOVA, Tukey’s post-hoc test, *p* < 0.05).

Previously we found that *recQ* mutations do not markedly impair *E*. *coli* culture growth under stress-free conditions [15]. In the present study we monitored cell growth in more frequent (5-min) *OD*_600_ (optical density at 600 nm) sampling intervals, allowing quantitative analysis of growth curves based on the modified Gompertz model (**Fig 1A, S1 Eq**) [21]. We also tested the effects of *recQ* mutations in the *recB1080* background (**Fig 1A, S1 Fig**). One-way ANOVA analysis of the maximal extent and rate of growth indicated that the *recB1080 recQ-dWH* double mutant and *recQ-dH* single-mutant strains had a slightly but significantly elevated growth extent and lowered rate, respectively, over several other assessed strains (**S1 Fig, S2 Table**). Apart from these findings, the analysis revealed no systematic effects of the mutations under these conditions.

To further probe potential defects in cell division, we determined the size distribution and morphology of cells by light microscopic analysis following methylene blue staining (**Fig 1B, Panel A in S2 Fig**). Strikingly, the *recB1080* mutation resulted in the appearance of large elongated cell structures (with areas larger than 4 μm^2^, compared to the average WT cell size around 1 μm^2^) that were not seen in strains with intact RecB protein. In all strains of *recB1080* background, about 15–20% of the total cell area, contributed by about 3–4% of the total number of cells, was found in these aberrant structures (**Fig 1C, Panel D in S2 Fig, S3 Table**). Aside from this effect, we found no significant differences between any strains regarding the average size of cell populations below 4 μm^2^ **(Panel B in S2 Fig and Panel C in S2 Fig, S3 Table**).

To assess whether the appearance of large cells in *recB1080*-based strains significantly influences the *OD*_600_ profiles (**Fig 1A**), we also determined the titer of colony forming units (CFU/ml) during growth of WT and *recB1080 ΔrecQ* strains (**Fig 1A inset**). We found no significant differences between the strains regarding growth rate and amplitude (**S1 Fig legend**), indicating that the *OD*_600_ curves are not markedly influenced by the observed differences in cell size distribution.

In summary, the above experiments show that *recQ* and *recB1080* mutations do not have systematic effects on cell culture growth under stress-free conditions. However, the *recB1080* mutation, but not *recQ* defects alone, selectively impairs the segregation of cells. The effects of *recB1080* and *recQ* mutations on cell growth and division are non-cumulative under stress-free conditions.

### RecQ and RecB functional defects exert cumulative effects on interstrand DNA crosslink repair

**Fig 2:**
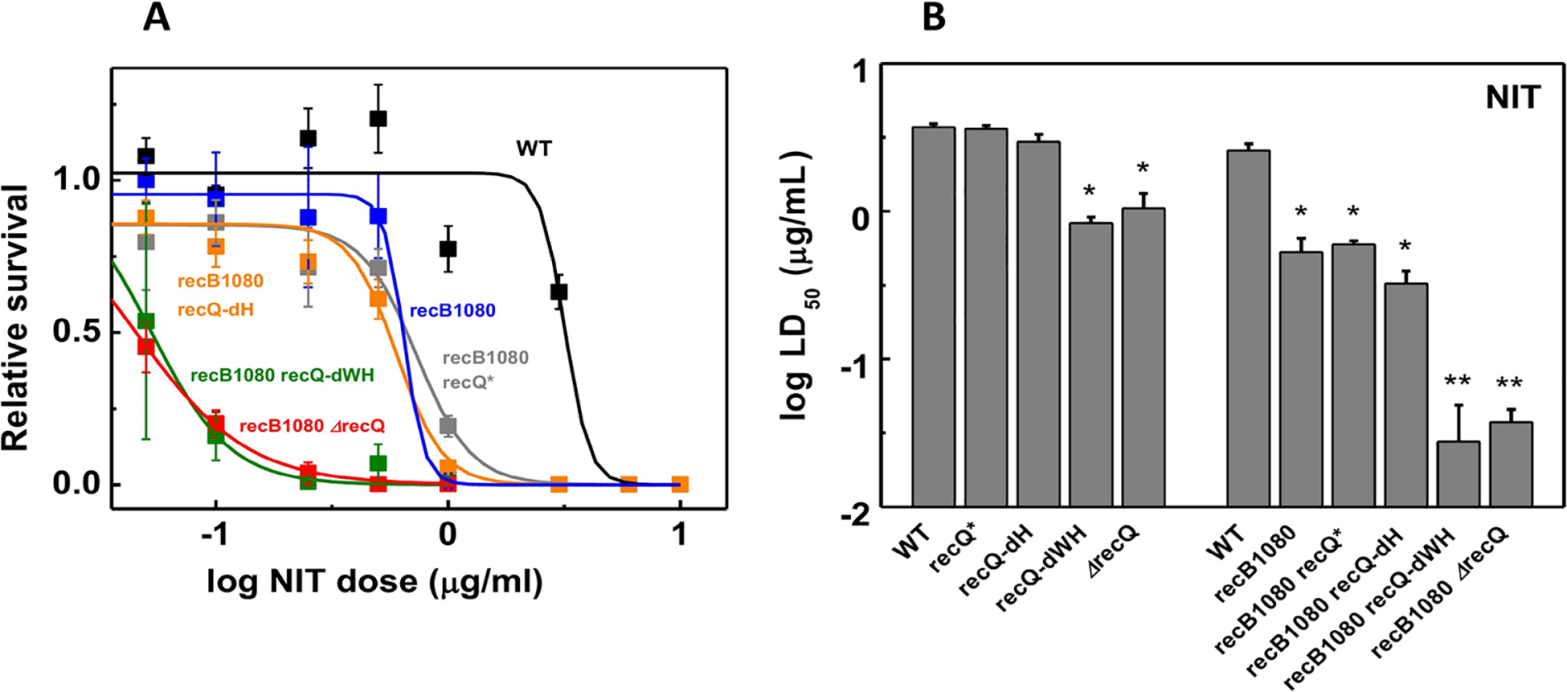
RecQ and RecB functional defects exert cumulative effects on the repair of nitrofurantoin-induced DNA damage. **(A)** Dose-dependent NIT survival of *E*. *coli* strains (means ± SE), fitted by a standard dose-response model (**S2 Eq**). Symbols for low survival values at high NIT doses (all measured up to 10 μg/mL) are mostly obscured by those of other strains with similar values. **(B)** Fitted log *LD*_50_ values of strains. Means ± SE of individual best-fits to biological replicates are shown. Asterisks indicate significant difference from the WT value (ANOVA, Tukey’s post-hoc test, *p* < 0.05). Double asterisks indicate further significant difference from values with single asterisk. Multiple WT controls are shown alongside mutant values determined in the same experimental run. Data for non-*recB1080* strains are from ref. [15]. Sample sizes and determined parameters are listed in **S3 Table**.

Nitrofurantoin (NIT) causes the formation of interstrand DNA crosslinks and oxidative DNA damage, and also inhibits translation in bacterial cells [22,23]. The *ΔrecQ* and *ΔrecB* mutations have been shown to sensitize *E*. *coli* cells to this antibiotic [24]. Previously we showed that the helicase-competent RecQ* and RecQ-dH constructs, but not the helicase-deficient RecQ-dWH protein, are capable of compensating NIT sensitivity to the WT level [15]. In the present study we found that the *recB1080* mutation causes NIT sensitization similar to that seen for *ΔrecQ* (**Fig 2**). Importantly, in the *recB1080* background, the *ΔrecQ* and *recQ-dWH* mutations (but not the dsDNA unwinding-competent recQ* and *recQ-dH* variants) caused significant further NIT sensitization (**Fig 2, S3 Table**). These results show that both the normal functioning RecBCD and the DNA unwinding activity of RecQ are necessary for efficient repair of NIT-induced DNA damage, and the effects of the functional deficiency of the two repair enzymes are cumulative.

### Loss of RecQ function further sensitizes *recB1080* cells to UV-induced lesions

**Fig 3:**
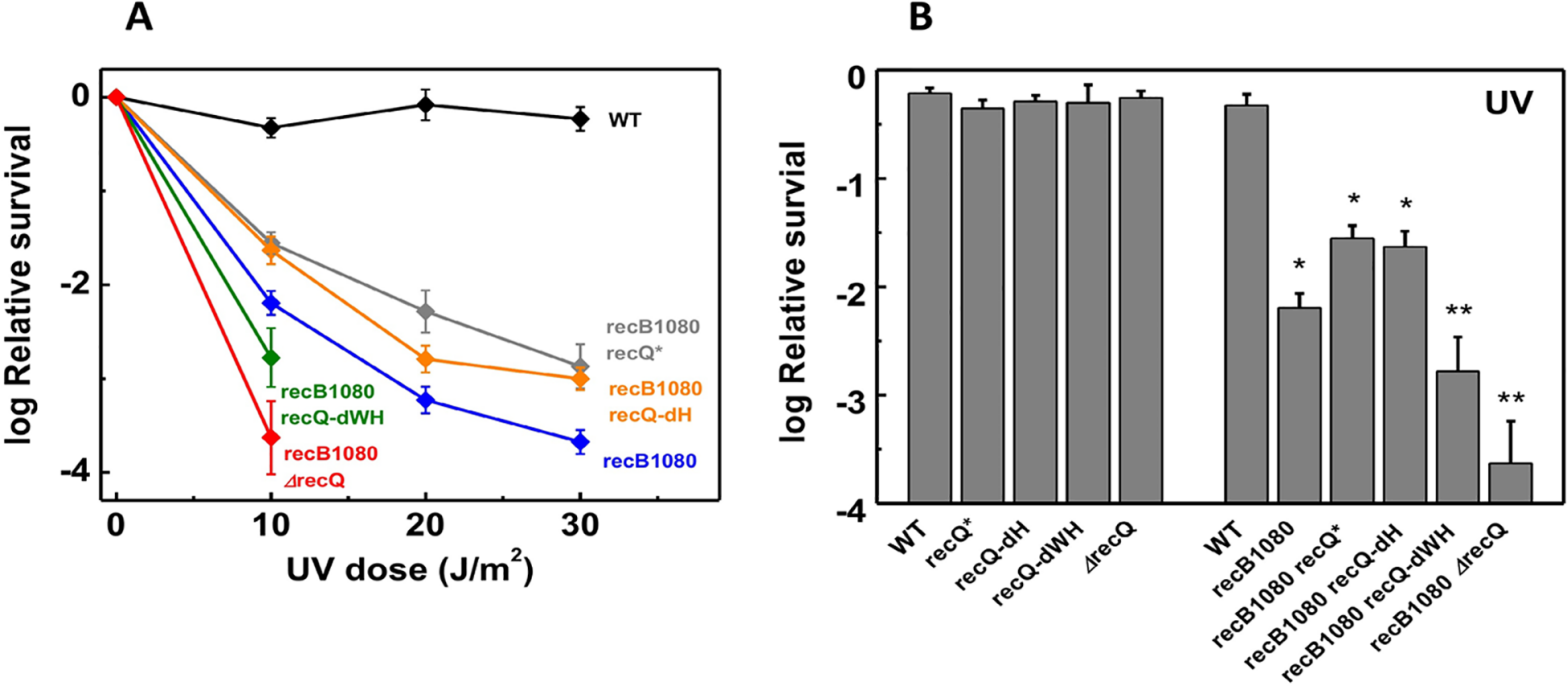
Loss of RecQ function further sensitizes *recB1080* cells to UV-induced lesions. **(A)** UV survival (mean ± SE) of *E*. *coli* strains. Means ± SE for *n* = 5 biological replicates are shown. Low survival values (< 10^−4^) for *recB1080 recQ-dWH* and *recB1080 ΔrecQ* strains at UV doses above 10 J/m^2^ could not be accurately determined. Due to this condition and the generally low UV survival values measured in the *recB1080* background, the standard dose-response model (**Fig 2B**) could not be applied. **(B)** Log relative survival values at 10 J/m^2^ UV irradiation (means ± SE). Asterisks indicate significant difference from the WT value (ANOVA, Tukey’s post-hoc test, *p* < 0.05). Double asterisks indicate further significant difference from each of the recB1080 recQ* and *recB1080 recQ-dH* strains (for *recB1080 recQ-dWH*), or from each of the *recB1080*, recB1080 recQ* and *recB1080 recQ-dH* strains (for *recB1080 ΔrecQ*). Multiple WT controls are shown alongside mutant values determined in the same experimental run. Data for non-*recB1080* strains are from ref.[15].

Previously we and others found that mutations in the *recQ* gene do not affect the sensitivity of *E*. *coli* cells to UV irradiation [5, 15, 25, 26]. In the present study we found that, contrary to RecQ deletion, the *recB1080* mutation drastically reduces the survival of *E*. *coli* cells upon UV radiation (**Fig 3**) in line with previous results [5,26]. Importantly, in the *recB1080* background, the lack of RecQ DNA unwinding activity (*ΔrecQ*, *recQ-dWH*) caused further reduction of UV survival, whereas this effect was not seen in strains expressing unwinding-competent RecQ constructs (recQ*, *recQ-dH*) (**Fig 3**). These results show that, in contrast to NIT sensitivity where the *recB1080* and *ΔrecQ* mutations individually have effects of the same magnitude (**Fig 2B**), normal RecBCD functions are more important than RecQ functions for the survival of UV-induced DNA lesions (**Fig 3B**). Nevertheless, the contribution of RecQ’s DNA unwinding activity to survival of UV-induced damage becomes manifest in the *recB1080* background (**Fig 3B**).

### RecQ defects compromise the survival of *recB1080* cells under replication stress

**Fig 4:**
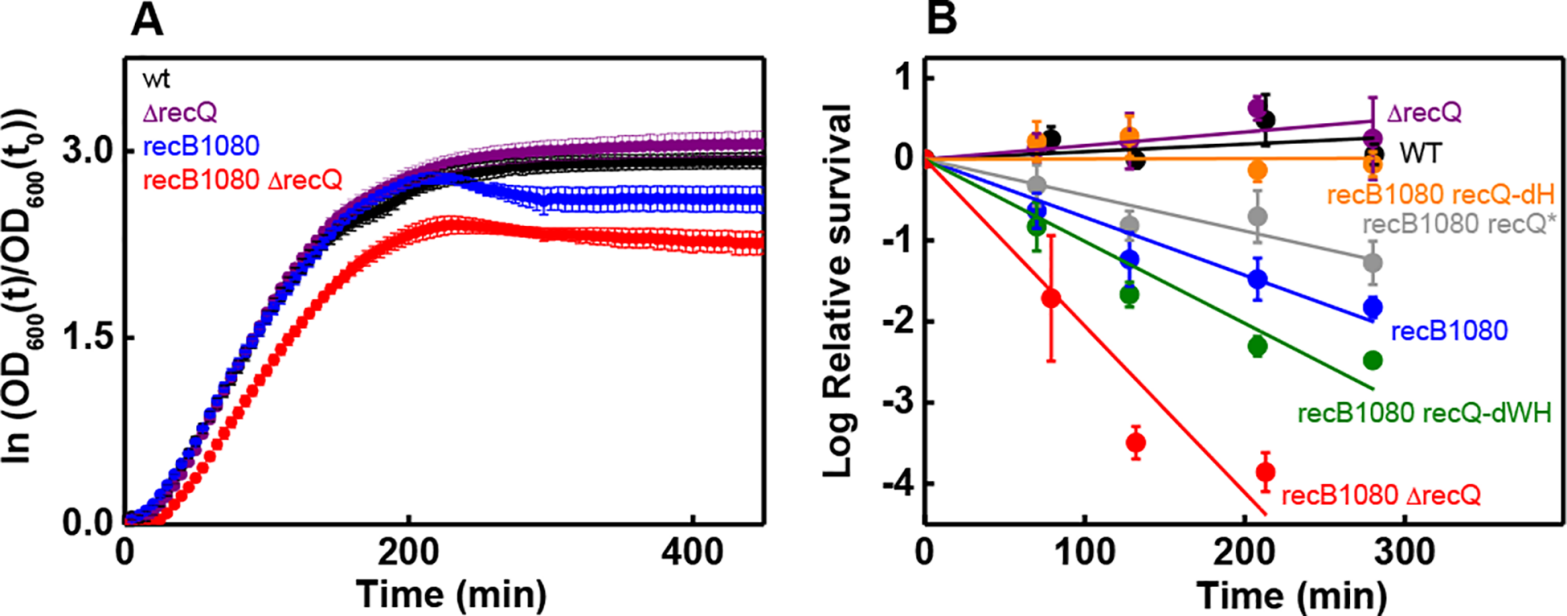
Loss of RecQ function compromises the survival of *recB1080* cells under replication stress. **(A)** Time-dependent *OD*_600_ profiles (ln (*OD*_600_(*t*)/*OD*_600_(*t*_0_)), means ± SE) of indicated *E*. *coli* strains in the presence of 100 mM HU. **(B)** Time-dependent HU (100 mM) survival profiles of indicated *E*. *coli* strains, monitored via changes in CFU/ml (means ± SE). Solid lines show linear regression best-fits to the time dependence of log-transformed relative survival data. Results of statistical comparison of best-fit slopes are shown in **S4 Table**.

To determine how RecBCD and RecQ functions contribute to cell growth under replication stress, we recorded the time-dependent *OD*_600_ profiles of *E*. *coli* strains in the presence of 100 mM hydroxyurea (HU) (**Fig 4A**). HU is known to deplete cellular dNTP pools and also to cause an increase in the intracellular concentration of reactive oxygen species (ROS) [27,28]. The *OD*_600_ profiles showed an initial increase even in the presence of HU, but a noticeable drop in *OD*_600_ was observed after 200 min in *recB1080*-based strains (**Fig 4A**). To assess the basis of this optical behavior, we determined the time-dependent survival of strains in 100 mM HU via monitoring CFU/ml counts, and applied a linear model for the time dependence of log-transformed relative survival values to aid statistical comparison between strains (**Fig 4B, S4 Table**). While both the WT and *ΔrecQ* strains showed stagnation over time, the *recB1080* single mutant and recB1080 recQ* double mutants showed a significant time-dependent reduction in survival. Interestingly, the *recB1080 recQ-dH* mutant a showed survival profile that was restored to the WT level. The survival of the *recB1080 recQ-dWH* and *recB1080 ΔrecQ* double mutants was even lower than that of other strains, indicated by statistically significant differences in most pairwise comparisons (**Fig 4B, S4 Table**). Taken together, the data indicate that, in contrast to the loss of RecQ helicase function alone, the *recB1080* mutation compromises cell survival under replication stress, and RecQ loss-of-function further sensitizes cells to replication stress in the *recB1080* background.

### RecQ helicase variants with enhanced processivity ameliorate *RecB1080*-induced impairment of recombination accuracy

The above results showed how *recB1080* and/or various *recQ* mutations affect cell survival, division and growth under stress-free and genome-damaging stress conditions. To quantify how the same mutations affect the accuracy of HR, we applied the well-established *Spi*^–^ λ phage assay that allows the determination of illegitimate recombination frequencies (IRF) [29]. Upon lytic induction, λ phage is normally excised from the genome of lysogenic *E*. *coli* strains by site-specific recombination. However, IR events produce phage variants that lack the *red* and *gam* genes and are therefore able to form plaques on P2 lysogenic *E*. *coli* strains (*Spi*^−^phenotype), thereby allowing their selection against WT phages.

**Fig 5:**
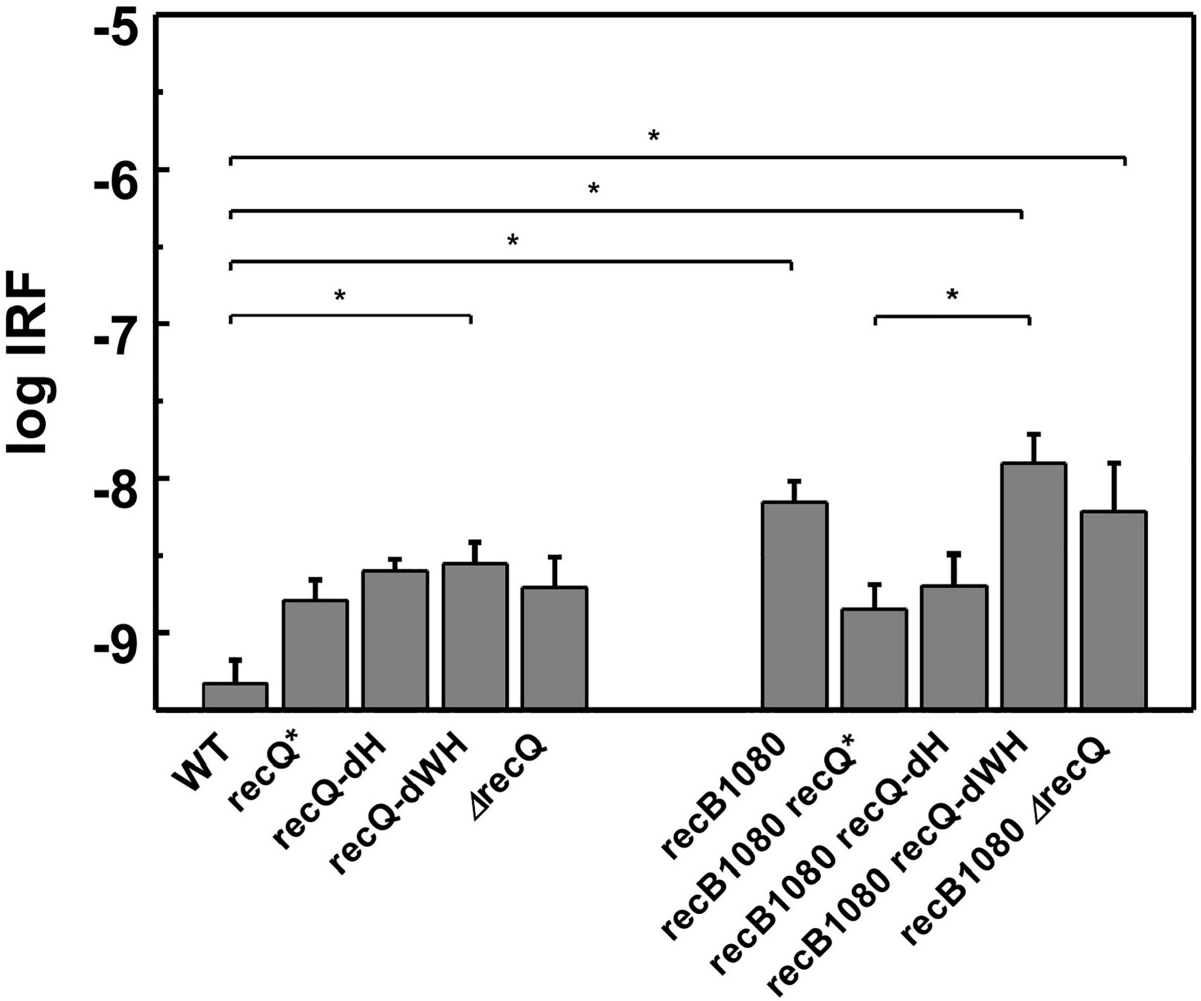
RecQ helicase variants with enhanced processivity ameliorate RecB1080-induced impairment of recombination accuracy. UV-free IRF values of *E*. *coli* strains (mean ± SE) are shown. Asterisks indicate significant pairwise differences (ANOVA, Tukey’s post-hoc test, *p* < 0.05). Data for non-*recB1080* strains are from ref. [15]. Determined parameters, sample sizes and statistical *p* values are listed in **S5 and S6 Tables**.

We performed IRF measurements on strains harboring WT and mutant *recQ* variants in the *recB1080* background under stress-free conditions, and synthesized the new results with our previous data on strains with WT *recB* background [15]. One-way ANOVA analysis (with Tukey’s post-hoc test) of data of all strains measured in the absence of UV irradiation showed significantly elevated IRF for the *recQ-dWH* and *recB1080* single-mutant, as well as for the *recB1080 recQ-dWH* and *recB1080 ΔrecQ* double mutant strains, compared to the WT strain (**Fig 5, S5 and S6 Tables**). (The IRF of *recB1080 recQ-dWH* was also significantly elevated over that of recB1080 recQ*.) Importantly, however, the IRF of the recB1080 recQ* and *recB1080 recQ-dH* double mutant strains was not significantly elevated over that of the WT strain (**Fig 5, S5 and S6 Tables**). This result indicates that the recQ* and *recQ-dH* mutations, resulting in RecQ helicase variants with enhanced DNA unwinding processivity but reduced invasion disruptase activity [15], suppress the elevated UV-free IRF phenotype of the *recB1080* mutation.

UV irradiation has been shown to cause a large increase in IRF [5, 8, 15]. Previously we found that, upon UV irradiation (50 J/m^2^), the recQ* and *recQ-dH* single mutant strains show significantly increased IRF as compared to the WT strain, suggesting that the enhanced DNA unwinding activity of the RecQ* and RecQ-dH proteins, in the absence of efficient invasion disruption, impairs HR accuracy under these conditions [15]. Unlike that for *recQ* mutants in WT *recB* background [15], the UV-induced IRF in *recB1080*-harboring strains could not be reliably determined due to the very low survival of these strains even at low UV doses (cf. **Fig 3**). A comparison of *ΔrecQ* and *recB1080* mutation-induced effects on IRF found in the present study with those in earlier reports is provided in SI (**S5 Table** legend).

Taken together, the IRF data reveal that in the absence of genomic stress (*i*.*e*., no UV irradiation), the aberrant RecB1080 activity leads to increased IRF. Strikingly, this IRF-enhancing effect of RecB1080 is abolished in double mutants expressing RecQ variants with enhanced helicase but compromised disruptase activities (recB1080 recQ*, *recB1080 recQ-dH*; **Fig 5, S5 and S6 Tables**)—even though the same RecQ variants in WT *recB* background caused increased IRF under UV-induced genomic stress leading to more frequently stalled replication and increased DSB formation (**S5 Table**) [15]. These results point to a functional antagonism between the applied specific RecBCD and RecQ mechanistic alterations (see below).

## Discussion

**Fig 6:**
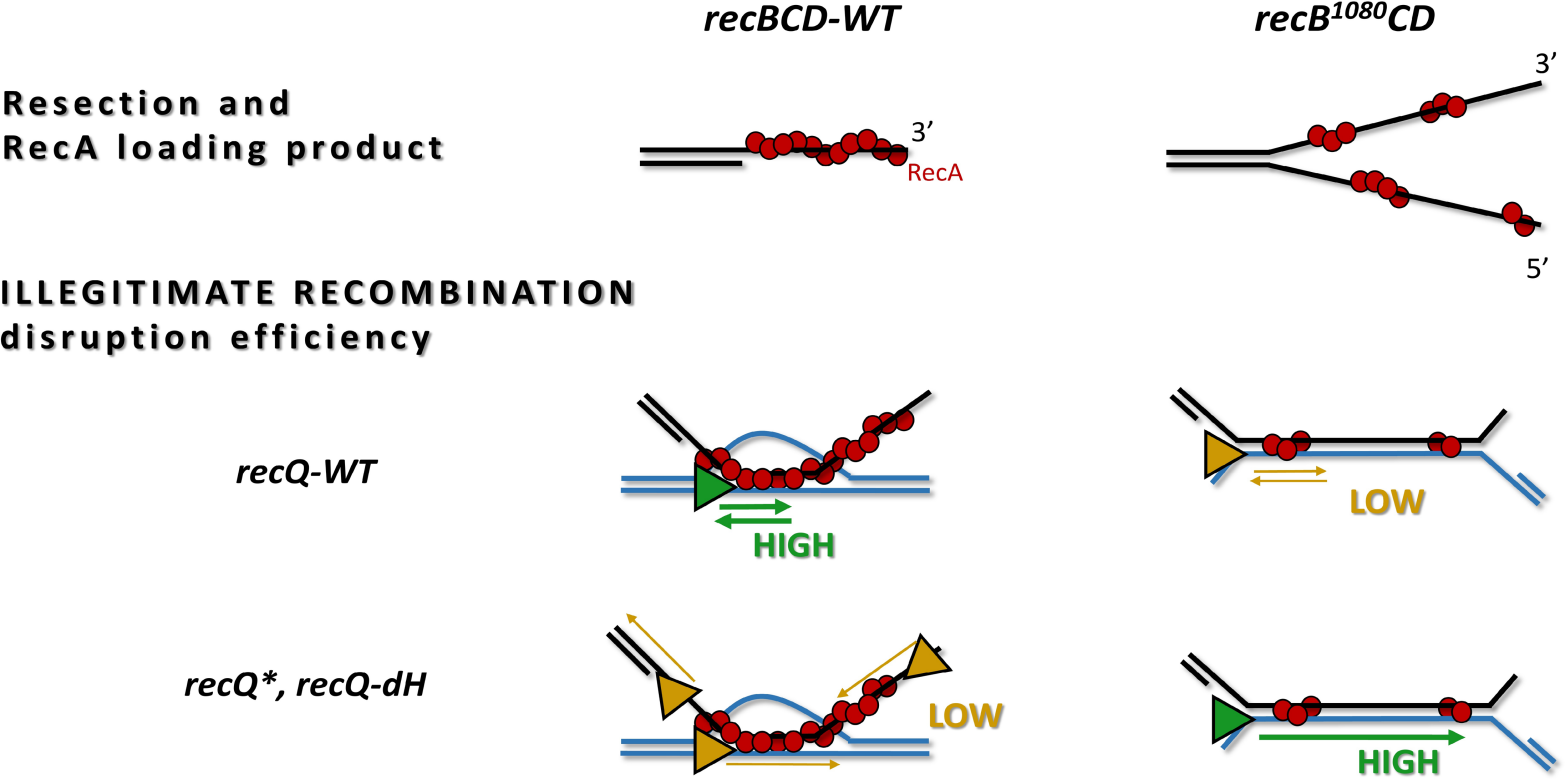
Functional fine-tuning between RecBCD and RecQ enzymes. **Upper row:** DSB processing by the RecBCD-WT complex (RecBCD protein not shown) results in RecA-loaded 3’ DNA overhangs. In contrast, RecB^1080^CD produces aberrant long, partially RecA-filled 5’ and 3’ overhangs. **Middle row:** The complex DNA unwinding, directed invasion disruption and shuttling activities of the RecQ-WT enzyme (triangle) enable efficient disruption of IR invasions (green) in the *recBCD-WT*, but low efficiency (yellow) in the *recB*^*1080*^*CD* background. **Lower row:** The enhanced (highly processive) DNA unwinding, but compromised directed invasion disruption and/or shuttling activities of RecQ* and RecQ-dH enzymes (triangle) are inefficient (yellow) for IR disruption in the *recBCD-WT* background. However, these activities become efficient (green) in the *recB*^*1080*^*CD* background where HR is more likely proceed through joint DNA molecules of aberrant structure.

Recent biochemical/biophysical investigations of the DNA-restructuring activities of RecBCD and RecQ enzymes yielded important insights into their mechanisms of action, and precisely defined the mechanistic perturbations characteristic of their engineered variants [5, 15, 25, 26, 30]. Specifically, the enhanced helicase but deficient nuclease and RecA-loading activities of the RecB^1080^CD complex lead to the formation of abnormally long, partially RecA-filled 5’ and 3’ DNA overhangs [6,31] (upper row in **Fig 6**), while the enhanced (highly processive) DNA unwinding but compromised DNA invasion disruption and/or shuttling activities of the RecQ* and RecQ-dH variants lead to impaired suppression of IR events [15]. In the present study, we quantitatively tested the *in vivo* cellular effect of these specific perturbations and the functional interplay between the above-mentioned genome maintenance enzymes.

Our data support that *recQ* mutations do not systematically affect *E*. *coli* cell growth under stress-free conditions, either in the *recB*-*WT* or in the *recB1080* background (**Fig 1, S1 Fig and S2 Fig, S2 and S3 Tables**). However, in line with earlier studies pointing to the role of RecBCD in late stages of replication and in the induction of DNA damage-dependent SOS response that halts cell division [32, 33], we detected significant filamentation in *recB1080* cells, which was unaffected by their *recQ* genotype (**Fig 1B and 1C, Panel A in S2 Fig, S3 Table**). The *recB1080* mutation was shown to cause a marked increase in the level of SOS induction even in the absence of genotoxic agents, whereas the *ΔrecQ* knockout did not influence SOS induction in either the *recB*-*WT* or the *recB1080* background [30]. Taken together, these results underscore the linkage between elevated SOS induction and filamentation (cell division defects) in *recB1080* cells. Thus, the resection of dsDNA ends, performed mainly by RecBCD in WT cells, appears to be catalyzed by the aberrant RecB^1080^CD enzyme in conjunction with the RecJ exonuclease in *recB1080* cells, while RecQ may only have a minor role in DSB resection. Accordingly, unlike *ΔrecQ*, the *recJ* knockout led to significantly lowered SOS induction in *recB1080* cells [30]. *recB1080*-induced SOS response and filamentation may reflect multiple possible underlying mechanisms. (*i*) Aberrant dsDNA end processing in *recB1080* cells may lead to aberrant RecA.ssDNA nucleoprotein structures that cannot initiate HR efficiently, and the persistence of these structures may trigger SOS response (30). (*ii*) The formation of aberrantly joined DNA molecules during HR-based replication restart, DSB repair or in late stages of replication, which cannot be resolved by RecQ helicase, may halt chromosome segregation and lead to filamentation. (*iii*) Due to its enhanced helicase activity, RecB^1080^CD may excessively unwind DNA structures, thereby facilitating aberrant DNA processing and, in turn, DNA damage.

We monitored cell survival or growth in the presence of three different genotoxic agents (NIT, UV irradiation, HU) to probe the interplay between RecBCD and RecQ activities during DNA damage repair (**Figs 2**–**4, S3 and S4 Tables**). NIT addition mostly causes interstrand DNA crosslinks, and also generates oxidative DNA damage [22,23]. UV irradiation predominantly leads to the formation of intrastrand DNA crosslinks and, to a lesser extent, 8-oxoguanine and oxidized pyrimidines [34]. Inter- and intrastrand crosslinks caused by NIT and UV, respectively, can both frequently lead to replication stalling and DSB formation [35,36]. HU inhibits ribonucleotide reductase and thus depletes cellular dNTP pools, causing general replicational stress. In addition, HU increases membrane permeability, leading to increased intracellular concentration of reactive oxygen species, causing additional DNA damage [27, 28]. Interestingly, the relative severity of the effects of *recB1080* mutation and RecQ DNA unwinding deficiency (*ΔrecQ*, *recQ-dWH*) were different in the case of different genome-damaging agents (**Figs 2**–**4, S3 and S4 Tables**). Both of the *recB1080* and *ΔrecQ* (or *recQ-dWH*) single mutations significantly sensitized cells to NIT (**Fig 2, S3 Table**). However, only *recB1080*, but not *ΔrecQ* or *recQ-dWH* alone, impaired the UV survival of cells (**Fig 3**, in line with ref. [5]). Similarly, unlike the *ΔrecQ* mutation, the *recB1080* mutation caused reduced survival in HU, and this effect was exacerbated by *recQ* loss-of-function in the *recB1080* background (**Fig 4, S4 Table**).

Two important general conclusions emerge from the above experiments. First, the effects of *recB1080* and RecQ deficiency (*ΔrecQ* or *recQ-dWH*) were mostly cumulative (i.e. double mutant phenotypes were generally more severe than any single mutant phenotype), showing that both the RecBCD and RecQ enzymes significantly contribute to various forms of DNA repair. Second, the RecQ* and RecQ-dH constructs fully compensated for RecQ-WT function in most cases, showing that shuttling during DNA unwinding and the directed invasion disruptase function (present in RecQ-WT, absent in RecQ* and RecQ-dH) [15] are dispensable in terms of the efficient repair of DNA lesions, either in the *recBCD-WT* or the *recB1080* background.

Interestingly, while *ΔrecQ* reduces UV survival in the *recB1080* (**Fig 3**; in line with ref. [5]) and also in the *ΔrecBCD* [37] background, *recQ* deficiency has no effect on UV survival in either *recBCD-WT* (**Fig 3**) or *recB1080 recD* [5] backgrounds where the RecA loading capability of the RecBCD complex is retained. Together these findings show that RecQ functions confer a significant advantage in DNA lesion repair only when the RecA loading function of RecBCD is compromised. These findings are in line with the genome instability phenotypes of RecQ helicase deficiencies in eukaryotes where, in the absence of a RecBCD homolog, the DSB resection and recombinase loading activities are not coupled in a single enzyme complex [12,38,39].

The *ΔrecB* mutation was found to significantly sensitize *E*. *coli* cells to HU in a *ruvABC-WT* dependent manner (27). The authors proposed that, upon replication fork stalling, the regression of the fork leads to the formation of a single Holliday junction in the absence of significant resection (*ΔrecB*), and this structure is then converted to a DSB by RuvC. In line with these earlier results, we found reduced HU survival for *recB1080*, as compared to the WT strain (further exacerbated by *recQ* loss-of-function, **Fig 4B**), indicating that compromised resection in the *recB1080* mutant strain leads to enhanced DSB formation.

Besides probing the efficiency of HR-based DNA repair via genotoxic agents, we also tested the interplay between RecBCD and RecQ activities in HR quality control, measured through the frequency of IR [29]. IR events have been proposed to occur through local base pairing between short homologous regions of non-allelic DNA sequences [8,29]. In line with earlier studies [5], we found that the *recB1080* mutation alone leads to increased IRF in the absence of UV irradiation (**Fig 5, S5 and S6 Tables**), presumably because at free DNA ends the RecB^1080^CD enzyme generates long, partially RecA-filled 5’ and 3’ ssDNA overhangs that are prone to form IR initiations (**Fig 6**). Intriguingly, however, we found that in the recB1080 recQ* and *recB1080 recQ-dH* double mutants, the IRF was not significantly higher than that in the WT strain (**Figs 5 and 6, S5 and S6 Tables**; see also below). The specific suppression of RecB1080-induced IR by RecQ* and RecQ-dH is remarkable in the light of the finding that the activities of the same RecQ variants lead to increased IRF (over WT) when DSB formation is facilitated by UV irradiation in the *recBCD-WT* background (**S5 Table**) [15]. Besides compromised IR suppression (**Fig 6**), these highly processive RecQ variants are likely to support enhanced DSB resection and RecA loading under these conditions.

Taken together, the above findings reveal a precise functional fine-tuning between HR initiation and quality control systems of WT *E*. *coli* cells, and provide an *in vivo* context for the characterized complex biochemical activities of the WT and mutant RecBCD and RecQ proteins (**Fig 6**) [5,15,20,26]. In the *recBCD-WT* background, the directed invasion disruption and shuttling activities of RecQ-WT enable efficient and selective disruption of IR invasions, whereas the enhanced DNA unwinding but compromised directed disruption and shuttling activities of RecQ* and RecQ-dH are counter-productive for IR suppression (**Fig 6**). However, RecQ-WT is less productive in disrupting IR events resulting from the aberrant long overhangs produced by RecB^1080^CD, while the aberrant RecQ* and RecQ-dH activities become productive for IR suppression in this context (**Fig 6**). RecBCD has thus been evolutionarily optimized for efficient resection/recombinase loading under normal conditions (low concentration of DNA ends), whereas the complex activities of RecQ are optimized for the suppression of harmful consequences of genomic stress in WT cells (imprecise HR at elevated DNA breakage/replication fork stalling). The fact that RecQ aberrations exert only mild effects on cell survival (short-term effect) but significantly compromise HR accuracy (long-term effect) is in accordance with the genome instability and cancer predisposition symptoms of human RecQ helicase deficiencies including Bloom’s, Werner’s, Rothmund-Thompson and RAPADILINO syndromes [13,39,40].

## Methods

### Bacterial strains and modifications

*recQ* and *recB* mutant strains (derivatives of MG1655 denoted as WT) were constructed using the Red/ET Recombination Kit (GeneBridges, Heidelberg, Germany) (**S1 Table**). Δr*ecQ* is a *recQ* knock-out mutant. In place of the WT *recQ* gene, the *recQ-dH* and *recQ-dWH* strains harbor *recQ* genes in which the regions 1569–1830 bp (resulting termination at aa 523) and 1239–1830 bp (resulting in termination at aa 413), respectively, have been deleted. In place of the respective WT enzymes, the recQ* and *recB1080* strains express the proteins harboring the Y555A and D1080A substitution, respectively. Point mutations were generated by QuikChange II XL site-directed mutagenesis (Agilent Technologies).

### Growth curves

*OD*_600_-based growth curves were recorded by inoculating aliquots of overnight *E*. *coli* clonal cultures into Luria-Bertani (LB) medium to a volume of 200 μl and *OD*_600_ = 0.01, followed by incubation at 37°C with aeration and monitoring their *OD*_600_ value in 5-min intervals in a Synergy H4 Hybrid Multi-Mode Microplate Reader instrument. Time-dependent changes in CFU/ml were followed by plating 5-μl aliquots of serial dilutions from cultures grown as described above. CFUs were counted after overnight growth on LB plates at 37°C. Curves were analyzed based on the modified Gompertz model (**S1 Eq**) [21].

### Cell staining and microscopy

For visualization, 10 μl of clonal cell cultures (OD_600_ = 0.7) were spread onto microscope slides, fixed in flame, stained with methylene blue (0.3 g/L) for five minutes, washed with distilled water and dried. Images were taken using a Nikon Japan Planflour microscope with objective 100x/1.30 Oil DIC H/N2. For cell area analysis, 10 images were randomly selected for each biological replicate (3 replicates for each genotype). Cell aggregates and overlapping cells were observed in regions of high cell density and in those around long filaments (present in all strains with *recB1080* background, cf. **A panel in S2 Fig and D panel in S2 Fig**). Images were converted to 8-bit grayscale images using ImageJ. Background correction of images was performed using the built-in IsoData automated threshold detection algorithm. Particle detection and area measurements were performed on background-corrected images using the built-in particle detection algorithm of ImageJ (0–infinity area detection, roundness range 0–1). Particles at image edges, as well as visually identified aggregates and overlapping cells were excluded from statistical analysis. Thus, the calculated cell area fractions occupied by large cells (**Fig 1C, S3 Table**) are slightly underestimated for strains where these structures appeared (*i*.*e*., for all strains with *recB1080* background). However, this condition does not affect the conclusion that the large cell fractions are significantly higher in each of these strains than in those with WT *recB* background.

### UV and NIT survival assays

In UV irradiation survival assays, cells were grown clonally to 4 x 10^8^ cells/ml at 37°C in LB medium. Aliquots of cultures were diluted, spread onto LB plates and irradiated with different UV doses (at 254 nm) using an UVP CX-2000 crosslinker. Following overnight growth at 37°C, colonies were counted to quantify survival. NIT survival assays were performed as described for UV survival assays, except that UV irradiation was omitted and cells were spread and grown on LB plates containing different concentrations of NIT.

### Hydroxyurea survival assays

These assays were performed as described for growth curve monitoring, except that 100 mM HU was included in the medium.

### λ Spi^-^ phage assay

The frequency of illegitimate recombination of λ phage was measured using the λ Spi^−^assay developed by Ikeda et al. [29]. For lysogenization, WT and mutant *E*. *coli* cells were grown to 7 x 10^8^ cells/ml at 30°C in T-broth (1 w/v % bacto tryptone (Difco), 0.5 w/v % NaCl, 10 mM MgSO_4_, 0.2 w/v % maltose). *λcI857* phage, isolated from HI1165 cells after heat shock induction of the lytic phase, was added to the cells (at multiplicity of infection = 2) and incubated for 30 min at 30°C. Cells were then spread onto T-plates (T-broth with 1.2 w/v % agar) and incubated at 30°C overnight. Single colonies were picked, amplified and checked for lysogeny by heat shock induction of the lytic phase (42°C for 15 min, followed by shaking (250 rpm) at 37°C and monitoring decrease in *OD*_600_). Three independent lysogens were generated for each genotype.

To analyze the formation of λ Spi^−^phages, lysogenic strains (**S1 Table**) were grown to 4 x 10^8^ cells/ml in λYP broth (1 w/v % bacto tryptone (Difco), 0.1 w/v % yeast extract (Oxoid), 0.25 w/v % NaCl, 0.15 w/v % NaHPO_4_, 0.018 w/v % MgSO_4_). To enter the lytic phase, *λcI857* prophage was heat induced by incubation of the culture at 42°C for 15 min. The culture was then incubated at 37°C for 2 hours with aeration, and centrifuged to isolate the phages from cell debris. The total phage titer and the titer of λ Spi^−^phages was determined by phage infection of YmeI and WL95 (P2 lysogen) *E*. *coli* cells, respectively (**S1 Table**). Mixtures of (diluted) aliquots of the phage suspension and YmeI or WL95 cells were incubated at room temperature for 30 min, mixed with λ top agar (1 w/v % bacto tryptone, 0.5 w/v % NaCl, 0.4 w/v % agar; for YmeI) or λ trypticase top agar (1 w/v % trypticase peptone (Difco), 0.5 w/v % NaCl, 0.4 w/v % agar; for WL95) and spread onto 1.2% agar plates (otherwise identical to the respective top agar). Plaques were counted after overnight incubation at 37°C. The burst size, calculated by dividing the titer of total phages by the titer of infective centers, was in the range of 18–145. The frequency of illegitimate recombination (IRF) was obtained by dividing the titer of λ Spi^−^phages by the titer of total phage.

### Data analysis and statistics

Mean ± SE values are reported in this study for the specified number of biological replicates (individual clones). Statistical significance (*p* < 0.05) was determined by ANOVA followed by the Tukey post-hoc test. For fitted parameters, statistical analysis was performed on the collection of best-fit values to individual datasets. UV survival and IRF values showed log-normal distribution, and thus the statistical analysis was performed on log-transformed values. Fitting and data analysis was performed using OriginLab 8.0 (Microcal Corp.).

## Supporting information

S1 Eq Modified Gompertz equation for bacterial growth curves (Fig 1A). (DOCX)

S2 Eq Standard dose-response equation used to fit NIT survival (Fig 2A). (DOCX)

S1 Fig Cell growth parameters determined in the absence of genomic stress. Parameters (panel **A**, growth amplitude (*A*); panel **B**, growth rate (μ_M_)) were determined from best-fits to individual growth curves, based on the modified Gompertz equation (**S1 Eq**). Means ± SE for *n* = 4–10 are indicated. Results of ANOVA analysis are summarized in **S2 Table**. Parameters determined from CFU counts (**Fig 1A inset**) were *A* = 3.18 ± 0.23 and μ_M_ = 0.013 ± 0.004 min^-1^ for WT, and *A* = 3.65 ± 0.33 and μ_M_ = 0.025 ± 0.013 min^-1^ for *recB1080 ΔrecQ* (differences between strains are not significant).
(TIF)

S2 Fig Cell size distribution of *E*. *coli* strains. **(A)** Example images of methylene blue stained cells.
**(B-C)** Cell size distribution histograms (area < 4 μm^2^) for *E*. *coli* strains.
**(D)** Scatter graph of cells with area > 4 μm^2^.
(TIF)

S1 Table E. coli strains used in this study. (PDF)

S2 Table Statistical *p* values of growth parameter comparisons. Shown are probability (*p*) values resulting from one-way ANOVA analysis (Tukey’s post-hoc test) of *A* and μ_m_ values (**S1 Eq**) obtained in best-fits to individual growth curves (averaged data shown in **Fig 1A**). Significant differences (*p* < 0.05) are highlighted in red.
(PDF)

S3 Table Morphological and NIT survival parameters. Significant difference from WT values is indicated by asterisks (one-way ANOVA, Tukey post-hoc test, *p* < 0.05). Double asterisk indicates further significant difference from values with single asterisk. Means ± SE values are shown for sample sizes (*n*, biological replicates (individual clones)) specified below. N.a., not applicable.
^a^ Gaussian means are shown for cell populations (± SE for *n* = 3 biological replicates) with individual cell area < 4 μm^2^, with the total number of analyzed cells in parentheses.
^b^ Specified as % of total cell area (± SE for *n* = 3 biological replicates) in cells with individual area > 4 μm^2^. Values in parentheses show the percentages for the number of large cells (> 4 μm^2^) within the assessed population.
^c^ Determined using **S2 Eq**. (*p* (Hill slope coefficient) values could not be robustly determined.) Statistics for individual best-fits to biological replicates are shown, with *n* in parentheses. Values for strains not harboring the *recB1080* mutation are from ref. [2] (**S1 Refs**).
^d^ WT control run in parallel with strains of *recB1080* background.
(PDF)

S4 Table Statistical *p* values for comparisons between HU survival profiles. Shown are probability (*p*) values resulting from one-way ANOVA analysis (Tukey’s post-hoc test) for the linear slopes of time-dependent log-transformed HU survival profiles (**Fig 4B**). Significant differences (*p* < 0.05) are highlighted in red.
(PDF)

S5 Table Illegitimate recombination frequencies (IRF). Values were determined by using the λ *Spi*^−^assay (3). Means ± SE are shown, with sample numbers (biological replicates) in parentheses. Results of one-way ANOVA analysis of UV-free log IRF values are summarized in **S6 Table**. Values for non-*recB1080* strains are from ref. [2] (**S1 Refs**). The UV-induced IRF values for recQ* and *recQ-dH* were significantly elevated (*p* < 0.05) over that of the WT strain [2] (**S1 Refs**).
N. d., not determined. Unlike those for *recQ* mutants in WT *recB* background [2] (**S1 Refs**), the UV-induced IRF values in *recB1080*-harboring strains could not be reliably determined due to the very low survival of these strains even at low UV doses (cf. **Fig 3**).
The magnitude and the cumulative nature of the IRF increase elicited by the *ΔrecQ* mutation and UV irradiation (~10-100-fold for each effect, ~100-1000-fold for the combined effect) was similar in our studies (this work and (2)) to those determined in earlier works [2–7] (**S1 Refs**). However, in the absence of UV irradiation, we found a larger effect for the *recB1080* mutation than that reported in ref. [7] (**S1 Refs**), and thus we observed no further IRF elevation in *recB1080 ΔrecQ*. We note that the mean IRF values determined in our studies are generally lower than those reported in the mentioned studies. This difference probably originates mostly from the fact that we applied the statistical analysis to log-transformed IRF values because the original values showed a log-normal spread. The applicability of this transformation is further substantiated by the fact that the average values collected from different studies themselves show a logarithmic spread. We also used higher sample numbers (*n* = 9–19) than those in previous studies (*n* = 2–4).
(PDF)

S6 Table Statistical *p* values of illegitimate recombination frequencies (log IRF values). Shown are probability (*p*) values resulting from one-way ANOVA analysis (Tukey’s post-hoc test) of log IRF values determined in the absence of UV irradiation (**Fig 5, S5 Table**). Significant differences (*p* < 0.05) are highlighted in red.
(PDF)

S1 Refs Supporting information references. (DOCX)
