# Supplementary material for: Functional fine-tuning between bacterial DNA recombination initiation and quality control systems
Source: PLoS One. 2018 Feb 22;13(2):e0192483. doi: 10.1371/journal.pone.0192483 (PMC5823372; doi:10.1371/journal.pone.0192483)
Supplement: S1 Eq — (DOCX) [file pone.0192483.s001.docx]

**S1 Eq. Modified Gompertz equation for bacterial growth curves (Fig 1A)**

$$y=Aexp\left\{ -exp\left[ \frac{\mu_{m}.e}{A}\left( \lambda-t \right)+1 \right] \right\}$$

Growth curves (**Fig 1A**) were fitted using the modified Gompertz equation [[1]](http://wizfolio.com/?citation=1&ver=3&ItemID=305&UserID=27292&AccessCode=639D22094DC14D00B6D2F8467E3BC4C8&CitationSuffix=) (**S1 Refs**), which has the advantage that the fitted parameters directly confer biological meanings. Growth (*y* = ln *OD*(*t*)/*OD*_0_) is plotted against time (*t*); *A* is the extent of maximal growth (ln *OD*_max_/*OD*_0_), *µ*_m_ is the maximum specific growth rate (expressed in reciprocal time dimension), and λ is the lag time (*t*-axis intercept of the tangent through the inflection point). Determined parameters are shown in **S1 Fig**. Lag times were not used for statistical analysis as they showed high variation, depending on inoculation conditions.
