# Supplementary material for: Functional fine-tuning between bacterial DNA recombination initiation and quality control systems
Source: PLoS One. 2018 Feb 22;13(2):e0192483. doi: 10.1371/journal.pone.0192483 (PMC5823372; doi:10.1371/journal.pone.0192483)
Supplement: S2 Eq — (DOCX) [file pone.0192483.s002.docx]

**S2 Eq. Standard dose-response equation used to fit NIT survival (Fig 2A)**

$$\text{Relative} \text{survival}=A_{0} + \frac{ A_{\max}- A_{0}}{1+10^{(\log LD_{50} - x) \cdot p}}$$

where *x* is the applied dose (log-transformed value); *A*_0_ and *A*_max_ are relative survival values at infinite and zero effector doses, respectively; *LD*_50_ is the dose that decreases relative survival to 50 %; and *p* is the Hill slope coefficient reflecting the steepness of the dose-response curve. Determined parameters are listed in **S3 Table**.
