# Supplementary figures and images for: Functional fine-tuning between bacterial DNA recombination initiation and quality control systems
Source: PLoS One. 2018 Feb 22;13(2):e0192483. doi: 10.1371/journal.pone.0192483 (PMC5823372; doi:10.1371/journal.pone.0192483)

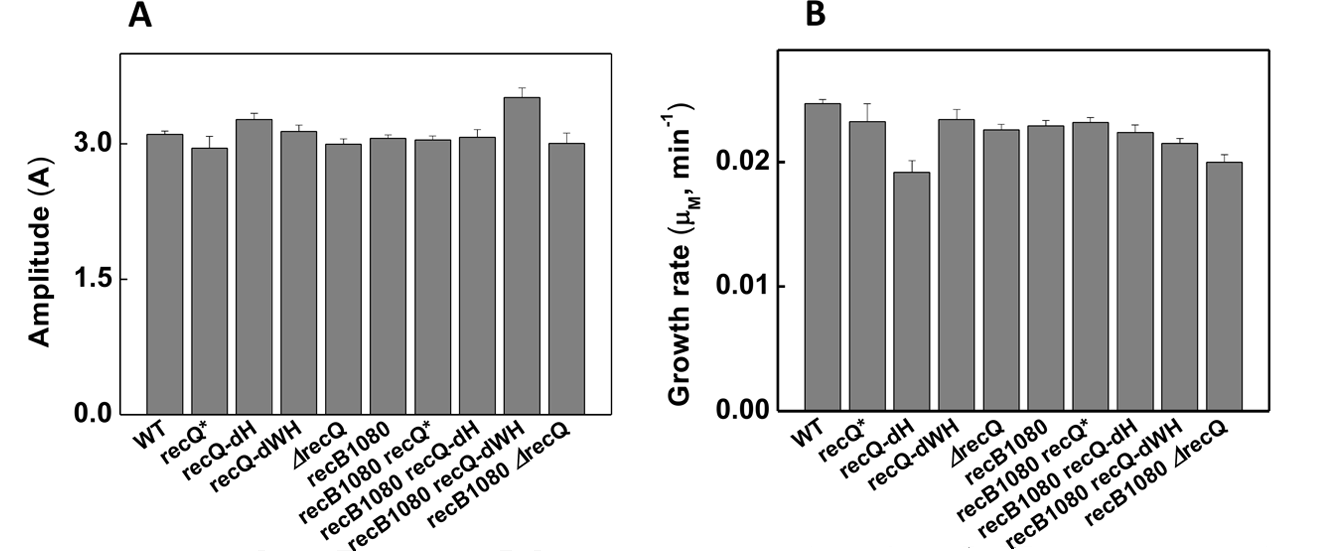

Supplement: S1 Fig — Parameters (panel A, growth amplitude (A); panel B, growth rate (μM)) were determined from best-fits to individual growth curves, based on the modified Gompertz equation (S1 Eq). Means ± SE for n = 4–10 are indicated. Results of ANOVA analysis are summarized in S2 Table. Parameters determined from CFU counts (Fig 1A inset) were A = 3.18 ± 0.23 and μM = 0.013 ± 0.004 min-1 for WT, and A = 3.65 ± 0.33 and μM = 0.025 ± 0.013 min-1 for recB1080 ΔrecQ (differences between strains are not significant). (TIF) [file pone.0192483.s003.tif]

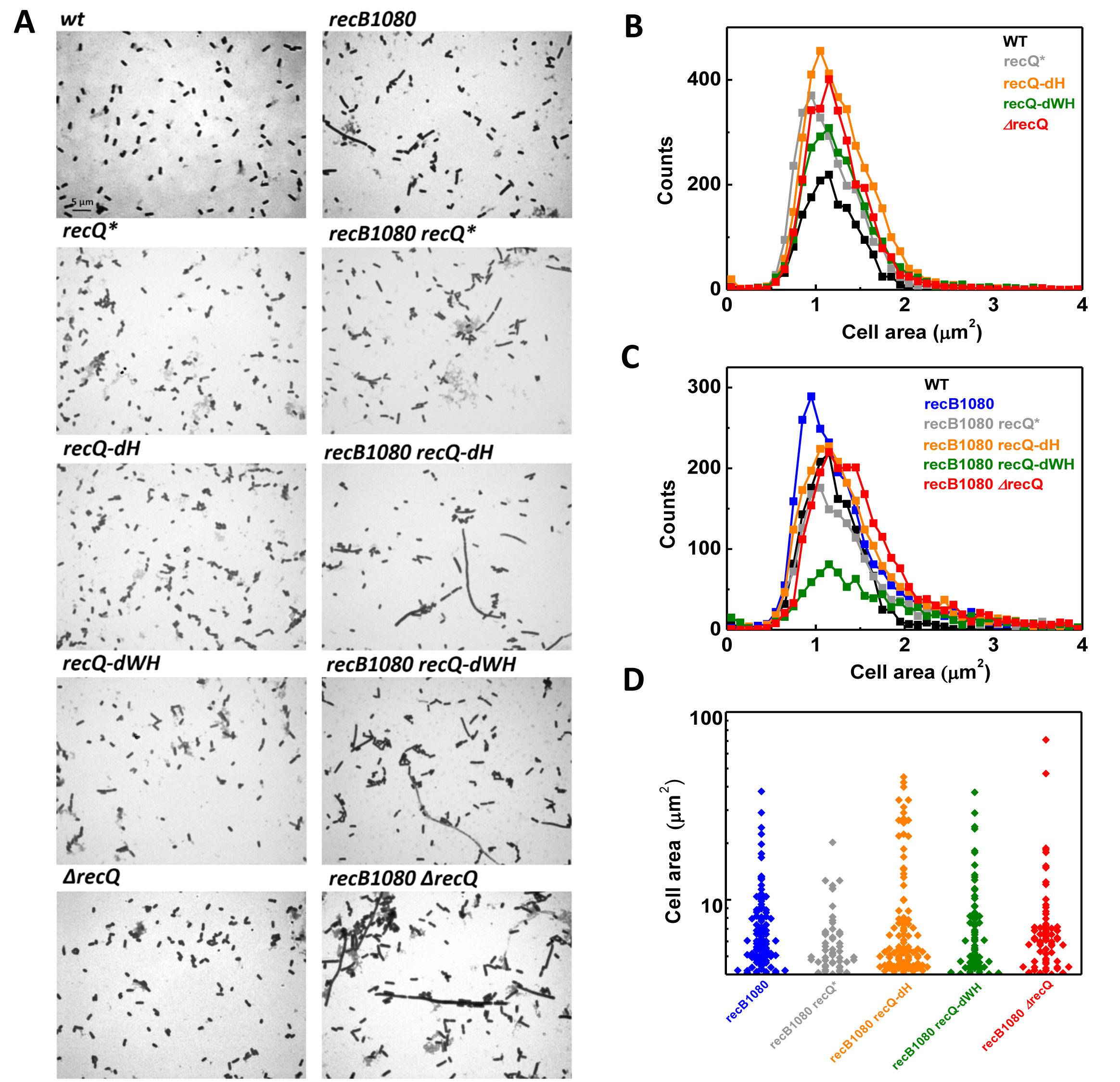

Supplement: S2 Fig — (A) Example images of methylene blue stained cells. (B-C) Cell size distribution histograms (area < 4 μm2) for E. coli strains. (D) Scatter graph of cells with area > 4 μm2. (TIF) [file pone.0192483.s004.tif]
