# Supplementary material for: Functional fine-tuning between bacterial DNA recombination initiation and quality control systems
Source: PLoS One. 2018 Feb 22;13(2):e0192483. doi: 10.1371/journal.pone.0192483 (PMC5823372; doi:10.1371/journal.pone.0192483)
Supplement: S1 Table — (PDF) [file pone.0192483.s005.pdf]

**Strain**	**Genotype**	**Source**
YmeI	*supE supF*	Ikeda laboratory
WL95	*supE supF metB trpR hsdR tonA (P2)*	Ikeda laboratory
HI1165	*λ cI857*	Ikeda laboratory
MG1655	F^- λ^- *ilvG*$^-$ *rfb-50 rph-1*	CGSC (Coli Genetic Stock Center)
MK1830	MG1655 *ΔrecQ* (*recQ* null mutant)	Our laboratory
MK1239	MG1655 *recQ-dWH* (1239-1830 bp deleted)	Our laboratory
MK1569	MG1655 *recQ-dH* (1569-1830 bp deleted)	Our laboratory
MK555	MG1655 *recQ** (Y555A substitution)	Our laboratory
MK1080R	MG1655 *recB1080*	Our laboratory
MK1830R	MG1655 *recB1080 ΔrecQ*	Our laboratory
MK1239R	MG1655 *recB1080 recQ-dWH*	Our laboratory
MK1569R	MG1655 *recB1080 recQ-dH*	Our laboratory
MK555R	MG1655 *recB1080 recQ**	Our laboratory
MK1080R*λ*	MG1655 *recB1080 λcI857*	Our laboratory
MK1830R*λ*	MG1655 *recB1080 ΔrecQ λcI857*	Our laboratory
MK1239R*λ*	MG1655 *recB1080 recQ-dWH λcI857*	Our laboratory
MK1569R*λ*	MG1655 *recB1080 recQ-dH λcI857*	Our laboratory
MK555R*λ*	MG1655 *recB1080 recQ* λcI857*	Our laboratory
