# Supplementary material for: Functional fine-tuning between bacterial DNA recombination initiation and quality control systems
Source: PLoS One. 2018 Feb 22;13(2):e0192483. doi: 10.1371/journal.pone.0192483 (PMC5823372; doi:10.1371/journal.pone.0192483)
Supplement: S2 Table — Shown are probability (p) values resulting from one-way ANOVA analysis (Tukey’s post-hoc test) of A and μm values (S1 Eq) obtained in best-fits to individual growth curves (averaged data shown in Fig 1A). Significant differences (p < 0.05) are highlighted in red. (PDF) [file pone.0192483.s006.pdf]

A

*recQ**	0.95								
recQ-dH	0.85	0.10							
recQ-dWH	1	0.86	0.96						
Δ recQ	1	1	0.24	0.97					
recB1080	1	0.99	0.61	1	1				
*recB1080 recQ**	1	0.999	0.49	1	1	1			
recB1080 recQ-dH	1	0.99	0.69	1	1	1	1		
recB1080 recQ-dWH	0.069	**2.49E-03**	0.50	0.13	**7.02E-03**	**3.00E-02**	**2.03E-02**	**3.86E-02**	
recB1080 Δ recQ	1.00	1	0.28	0.98	1	1	1	1	**8.69E-03**
	WT	*recQ**	*recQ-dH*	*recQ-dWH*	*Δ recQ*	*recB1080*	*recB1080 recQ**	*recB1080 recQ-dH*	*recB1080 recQ-dWH*

μ_M

*recQ**	0.97								
recQ-dH	**1.97E-04**	**1.32E-02**							
recQ-dWH	0.99	1	**8.59E-03**						
Δ recQ	0.78	1	0.07	1					
recB1080	0.99	1	**3.01E-02**	1	1				
*recB1080 recQ**	0.96	1	**1.52E-02**	1	1	1			
recB1080 recQ-dH	0.67	1	0.11	1	1	1	1		
recB1080 recQ-dWH	0.32	0.94	0.58	0.90	1	0.98	0.95	1	
recB1080 Δ recQ	**1.28E-02**	0.22	1	0.17	0.52	0.35	0.24	0.64	0.97
	WT	*recQ**	*recQ-dH*	*recQ-dWH*	*Δ recQ*	*recB1080*	*recB1080 recQ**	*recB1080 recQ-dH*	*recB1080 recQ-dWH*
