# Supplementary material for: Functional fine-tuning between bacterial DNA recombination initiation and quality control systems
Source: PLoS One. 2018 Feb 22;13(2):e0192483. doi: 10.1371/journal.pone.0192483 (PMC5823372; doi:10.1371/journal.pone.0192483)
Supplement: S3 Table — Significant difference from WT values is indicated by asterisks (one-way ANOVA, Tukey post-hoc test, p < 0.05). Double asterisk indicates further significant difference from values with single asterisk. Means ± SE values are shown for sample sizes (n, biological replicates (individual clones)) specified below. N.a., not applicable. a Gaussian means are shown for cell populations (± SE for n = 3 biological replicates) with individual cell area < 4 μm2, with the total number of analyzed cells in parentheses. b Specified as % of total cell area (± SE for n = 3 biological replicates) in cells with individual area > 4 μm2. Values in parentheses show the percentages for the number of large cells (> 4 μm2) within the assessed population. c Determined using S2 Eq. (p (Hill slope coefficient) values could not be robustly determined.) Statistics for individual best-fits to biological replicates are shown, with n in parentheses. Values for strains not harboring the recB1080 mutation are from ref. [2] (S1 Refs). d WT control run in parallel with strains of recB1080 background. (PDF) [file pone.0192483.s007.pdf]

Genotype	Cell area (µm^2) [a]	Fraction of large cells (%) [b]	NIT log LD_{50} (mg/L) [c]
WT	1.09 ± 0.10 (1595)	0	0.57 ± 0.02 (14)
*recQ**	1.07 ± 0.06 (2745)	0	0.56 ± 0.02 (15)
recQ-dH	1.21 ± 0.07 (3717)	0.3 ± 0.2 (0.1 ± 0.05)	0.47 ± 0.05 (10)
recQ-dWH	1.18 ± 0.07 (2546)	0.7 ± 0.4 (0.2 ± 0.08)	-0.08 ± 0.04 (10)*
ΔrecQ	1.18 ± 0.05 (2888)	0.6 ± 0.3 (0.1 ± 0.06)	0.02 ± 0.10 (11)*
WT [d]	n.a.	n.a.	0.50 ± 0.01 (3)
recB1080	1.09 ± 0.02 (2529)	18.6 ± 5.6* (3.8 ± 1.3)*	-0.50 ± 0.18 (4)*
*recB1080 recQ**	1.15 ± 0.09 (1701)	12.2 ± 1.5* (3.1 ± 0.6)*	-0.23 ± 0.03 (9)*
recB1080 recQ-dH	1.21 ± 0.10 (2418)	22.5 ± 4.6* (4.0 ± 0.6)*	-0.34 ± 0.03 (9)*
recB1080 recQ-dWH	1.25 ± 0.09 (1004)	15.0 ± 2.0* (3.7 ± 1.4)*	-1.56 ± 0.25 (4)**
recB1080 ΔrecQ	1.32 ± 0.05 (2354)	14.0 ± 3.7* (2.9 ± 0.5)	-1.43 ± 0.09 (5)**
