# Supplementary material for: Functional fine-tuning between bacterial DNA recombination initiation and quality control systems
Source: PLoS One. 2018 Feb 22;13(2):e0192483. doi: 10.1371/journal.pone.0192483 (PMC5823372; doi:10.1371/journal.pone.0192483)
Supplement: S4 Table — Shown are probability (p) values resulting from one-way ANOVA analysis (Tukey’s post-hoc test) for the linear slopes of time-dependent log-transformed HU survival profiles (Fig 4B). Significant differences (p < 0.05) are highlighted in red. (PDF) [file pone.0192483.s008.pdf]

ΔrecQ	0.99					
recB1080	**<1E-04**	**<1E-04**				
*recB1080 recQ**	**0.0012**	**0.0002**	0.24			
recB1080 recQ-dH	0.98	0.78	**<1E-04**	**0.0085**		
recB1080 recQ-dWH	**<1E-04**	**<1E-04**	0.16	**0.0005**	**<1E-04**	
recB1080 ΔrecQ	**<1E-04**	**0.0001**	**<1E-04**	**<1E-04**	**<1E-04**	**<1E-04**
	WT	*ΔrecQ*	*recB1080*	*recB1080 recQ**	*recB1080 recQ-dH*	*recB1080 recQ-dWH*
