# Supplementary material for: Functional fine-tuning between bacterial DNA recombination initiation and quality control systems
Source: PLoS One. 2018 Feb 22;13(2):e0192483. doi: 10.1371/journal.pone.0192483 (PMC5823372; doi:10.1371/journal.pone.0192483)
Supplement: S5 Table — Values were determined by using the λ Spi−assay (3). Means ± SE are shown, with sample numbers (biological replicates) in parentheses. Results of one-way ANOVA analysis of UV-free log IRF values are summarized in S6 Table. Values for non-recB1080 strains are from ref. [2] (S1 Refs). The UV-induced IRF values for recQ* and recQ-dH were significantly elevated (p < 0.05) over that of the WT strain [2] (S1 Refs). N. d., not determined. Unlike those for recQ mutants in WT recB background [2] (S1 Refs), the UV-induced IRF values in recB1080-harboring strains could not be reliably determined due to the very low survival of these strains even at low UV doses (cf. Fig 3). The magnitude and the cumulative nature of the IRF increase elicited by the ΔrecQ mutation and UV irradiation (~10-100-fold for each effect, ~100-1000-fold for the combined effect) was similar in our studies (this work and (2)) to those determined in earlier works [2–7] (S1 Refs). However, in the absence of UV irradiation, we found a larger effect for the recB1080 mutation than that reported in ref. [7] (S1 Refs), and thus we observed no further IRF elevation in recB1080 ΔrecQ. We note that the mean IRF values determined in our studies are generally lower than those reported in the mentioned studies. This difference probably originates mostly from the fact that we applied the statistical analysis to log-transformed IRF values because the original values showed a log-normal spread. The applicability of this transformation is further substantiated by the fact that the average values collected from different studies themselves show a logarithmic spread. We also used higher sample numbers (n = 9–19) than those in previous studies (n = 2–4). (PDF) [file pone.0192483.s009.pdf]

Genotype	log IRF (UV-free)	log IRF (UV, 50 J/m^2)
WT	-9.33 ± 0.15 (19)	-7.58 ± 0.16 (17)
*recQ**	-8.79 ± 0.13 (10)	-6.75 ± 0.18 (11)
recQ-dH	-8.60 ± 0.08 (10)	-6.33 ± 0.28 (11)
recQ-dWH	-8.55 ± 0.14 (15)	-7.09 ± 0.21 (11)
ΔrecQ	-8.71 ± 0.20 (14)	-6.91 ± 0.13 (13)
recB1080	-8.16 ± 0.14 (11)	n.d.
*recB1080 recQ**	-8.85 ± 0.16 (11)	n.d.
recB1080 recQ-dH	-8.70 ± 0.20 (11)	n.d.
recB1080 recQ-dWH	-7.90 ± 0.18 (9)	n.d.
recB1080 ΔrecQ	-8.21 ± 0.31 (11)	n.d.
