# Supplementary material for: Functional fine-tuning between bacterial DNA recombination initiation and quality control systems
Source: PLoS One. 2018 Feb 22;13(2):e0192483. doi: 10.1371/journal.pone.0192483 (PMC5823372; doi:10.1371/journal.pone.0192483)
Supplement: S6 Table — Shown are probability (p) values resulting from one-way ANOVA analysis (Tukey’s post-hoc test) of log IRF values determined in the absence of UV irradiation (Fig 5, S5 Table). Significant differences (p < 0.05) are highlighted in red. (PDF) [file pone.0192483.s010.pdf]

*recQ**	0.47								
recQ-dH	0.10	1							
recQ-dWH	**1.83E-02**	1	1						
Δ recQ	0.15	1	1	1					
recB1080	**1.29E-04**	0.39	0.84	0.85	0.48				
*recB1080 recQ**	0.59	1	1	0.97	1	0.24			
recB1080 recQ-dH	0.21	1	1	1	1	0.59	1		
recB1080 recQ-dWH	**6.34E-06**	0.07	0.32	0.30	0.09	1	**3.41E-02**	0.14	
recB1080 Δ recQ	**3.36E-04**	0.53	0.92	0.94	0.63	1	0.35	0.73	0.98
	WT	*recQ**	*recQ-dH*	*recQ-dWH*	*Δ recQ*	*recB1080*	*recB1080 recQ**	*recB1080 recQ-dH*	*recB1080 recQ-dWH*
