# Supplementary material for: Functional fine-tuning between bacterial DNA recombination initiation and quality control systems
Source: PLoS One. 2018 Feb 22;13(2):e0192483. doi: 10.1371/journal.pone.0192483 (PMC5823372; doi:10.1371/journal.pone.0192483)
Supplement: S1 Refs — (DOCX) [file pone.0192483.s011.docx]

**Supplementary references**

1. Zwietering MH, Jongenburger I, Rombouts FM, van 't Riet K. Modeling of the bacterial growth curve. Appl Environ Microbiol. 1990;56(6):1875-81.

2. Harami GM, Seol Y, In J, Ferencziová V, Martina M, Gyimesi M et al. Shuttling along DNA and directed processing of D-loops by RecQ helicase support quality control of homologous recombination. Proceedings of the National Academy of Sciences. 2017;114,(4,):E466-75.

3. Ikeda H, Shimizu H, Ukita T, Kumagai M. A novel assay for illegitimate recombination in Escherichia coli: stimulation of lambda bio transducing phage formation by ultra-violet light and its independence from RecA function. (1). Adv Biophys. 1995;31:197-208.

4. Hanada K, Iwasaki M, Ihashi S, Ikeda H. UvrA and UvrB suppress illegitimate recombination: synergistic action with RecQ helicase. Proc Natl Acad Sci U S A. 2000;97(11):5989-94.

5. Hanada K, Ukita T, Kohno Y, Saito K, Kato J, Ikeda H. RecQ DNA helicase is a suppressor of illegitimate recombination in Escherichia coli. Proc Natl Acad Sci U S A. 1997;94(8):3860-5.

6. Janscak P, Garcia PL, Hamburger F, Makuta Y, Shiraishi K, Imai Y et al. Characterization and Mutational Analysis of the RecQ Core of the Bloom Syndrome Protein. J Mol Biol. 2003;330(1):29-42.

7. Ivanković S, Đermić D. DNA end resection controls the balance between homologous and illegitimate recombination in Escherichia coli. PloS one. 2012;7(6):e39030.
